# Pouch-Type Asymmetric Supercapacitor Based on Nickel–Cobalt Metal–Organic Framework

**DOI:** 10.3390/ma16062423

**Published:** 2023-03-17

**Authors:** Surya. V. Prabhakar Vattikuti, Nguyen To Hoai, Jie Zeng, Rajavaram Ramaraghavulu, Nam Nguyen Dang, Jaesool Shim, Christian M. Julien

**Affiliations:** 1 School of Mechanical Engineering, Yeungnam University, 280 Daehak-Ro, Gyeongsan 712-749, Gyeongbuk, Republic of Korea; 2 Future Materials & Devices Laboratory, Institute of Fundamental and Applied Sciences, Duy Tan University, Ho Chi Minh City 700000, Vietnam; 3 The Faculty of Environmental and Chemical Engineering, Duy Tan University, Da Nang 550000, Vietnam; 4 Department of Physics, School of Applied Sciences, REVA University, Bangalore 560064, India; 5 Institut de Minéralogie, de Physique des Matériaux et de Cosmochimie (IMPMC), Sorbonne Université, CNRS-UMR 7590, 4 Place Jussieu, 75252 Paris, France

**Keywords:** energy storage, electrode materials, MOFs, supercapacitor, hierarchical structure

## Abstract

Bimetal–organic frameworks (BMOFs) have attracted considerable attention as electrode materials for energy storage devices because of the precise control of their porous structure, surface area, and pore volume. BMOFs can promote multiple redox reactions because of the enhanced charge transfer between different metal ions. Therefore, the electroactivity of the electrodes can be significantly improved. Herein, we report a NiCo-MOF (NCMF) with a three-dimensional hierarchical nanorod-like structure prepared using a facile solvo-hydrothermal method. The as-prepared NCMF was used as the positive electrode in a hybrid pouch-type asymmetric supercapacitor device (HPASD) with a gel electrolyte (KOH+PVA) and activated carbon as the negative electrode. Because of the matchable potential windows and specific capacitances of the two electrodes, the assembled HPASD exhibits a specific capacitance of 161 F·g^−1^ at 0.5 A·g^−1^, an energy density of 50.3 Wh·kg^−1^ at a power density of 375 W·kg^−1^, and a cycling stability of 87.6% after 6000 cycles. The reported unique synthesis strategy is promising for producing high-energy-density electrode materials for supercapacitors.

## 1. Introduction

The rapid development of electric vehicles and the widespread use of portable electronic devices has increased the need for energy storage devices with high energy densities and long cycle life. Primary energy storage devices, such as supercapacitors and batteries, have their advantages and disadvantages. Supercapacitors offer high power density and long cycle life but low energy density [1]. At the same time, traditional energy storage devices cannot meet all the application requirements. In recent years, asymmetric supercapacitors (ASCs) have attracted significant attention by combining the advantages of batteries and supercapacitors, thereby allowing them to fulfill the requirements of powering electric vehicles and other multifunctional electronic products [1,2]. Therefore, the development of such ASCs has emerged as the focus of supercapacitor research. However, identifying suitable electrode materials for asymmetric devices is challenging because such materials must not only exhibit good electrochemical activity but should also possess comparable characteristics. Thus far, in most studies on asymmetric supercapacitors, electrodes have been produced from various metal oxides through rational design. Generally, activated carbon (AC) is used as a negative electrode. The performance of such asymmetric devices is not ideal [3]; therefore, new well-matched electrodes need to be developed to realize ASCs with high energy densities.

Metal–organic frameworks (MOFs), which are known as porous coordination polymers, are a new class of crystalline porous materials with periodic network structures generated by organic ligands and metal-ion clusters. Owing to their tailored structures, MOFs have large surface areas and multifunctional properties [4]. However, bare MOFs generally exhibit low electrical conductivity, weak structural flexibility, and steric hindrance for ion insertion; therefore, single-component MOFs are not suitable for application in energy storage devices. However, MOFs may be converted into bimetal–organic frameworks (BMOFs) using appropriate redox reactions. Compared with monometallic MOFs, mixed-metal–organic frameworks or BMOFs exhibit better electrochemical performance because of the enhanced charge transfer between the metal ions [5]. Owing to their inherent advantages, MOFs have potential applications in many scientific fields. For example, nickel- or cobalt-based MOFs exhibit good capacitance owing to their abundant redox reactions, large electrolyte-accessible area, and high electrochemical conductivity, which enable numerous active centers and fast charge transfer [6]. Therefore, nanostructures based on bimetallic or hybrid MOFs are more promising than single-component MOFs for further enhancing the energy storage capacity of electrochemical devices.

The enhanced energy storage of BMOF nanostructures is attributed to changes in the local coordination environment and electronic structure owing to the incorporation of metal cations [7,8]. Additionally, bimetallic species in MOFs enhance electrical conductivity and redox chemical rates because of their multiple oxidation states. Furthermore, they can act as electron mediators to accelerate charge transfer to metals through organic linkers, thereby enhancing the energy storage capacity. However, the morphology of MOFs is also crucial for the performance of hybrid devices. MOF-based materials possess hierarchical nanostructures with enhanced capacitance and rate performance because the abundant redox active sites provide multiple electron transport pathways. Therefore, recent studies focus on the rational design of hierarchical redox-based MOFs with controllable geometries for enhanced electrochemical activity.

Xiao et al. [9] developed a BMOF composed of Ni and Co metal ions with a 12 benzene-1,4-dicarboxylate (BDC) linker, which exhibited the highest specific capacitance of 1300 F·g^−1^ at 1 A·g^−1^ with good cycling stability (71% after 3000 cycles). Tian et al. [10] developed flower-like nanosheet Ni-based BMOFs (composed of Zn, Cu, Fe, or Co) grown on electrospun nanofibers. Among these BMOFs, NF@Co-Ni-MOF exhibited the highest specific capacitance (1096 F·g^−1^ at 1 A·g^−1^). Furthermore, when used as the positive electrode (with reduced graphene oxide (rGO) as the negative electrode) in hybrid devices, it exhibited the highest energy density (*E*_*d*_) of 94 W·kg^−1^ at a power density (*P_d_*) of 1600 W·kg^−1^ with good cycling stability up to 10,000 cycles. He et al. [11] demonstrated an *E_d_* of 28.6 W·kg^−1^ at a *P_d_* of 100 W·kg^−1^ for HPASD with BMOF-derived ZnCo_2_O_4_ as a positive electrode and nanoporous carbon obtained via carbonization with HCl at 900 °C as the negative electrode. Radhika et al. [12] reported a Ni/Co-MOF ACS electrode synthesized by a facile hydrothermal method using trimesic acid as a structure directing linker, which exhibited a high specific capacitance of 2041 F·g^−1^ at a scan rate of 2 mV·s^−1^ and 980 F·g^−1^ at a current density of 2.5 A·g^−1^. Kurisingal et al. [13] showed that the aqueous synthesis of a bimetallic MOF with Ni and Co as the active metal centers and benzene-1,4-dicarboxylic acid as the linker has been achieved rapidly in high yield using microwave irradiation. The BET surface area and pore volume of Ni–Co MOF are 50.8 m^2^·g^−1^ and 0.183 cm^3^·g^−1^, respectively. Hong and co-workers [14] fabricated nanostructured Ni-Co-MOF/graphene oxide composites as capacitor electrodes using 2-methylimidazole as an inexpensive organic ligand. At a current density of 1 A·g^−1^, the maximum specific capacitance was 447.2 F·g^−1^. A Ni/Co-based bimetallic MOF [CoNi(µ_3_-tp)_2_(m_2_-pyz)_2_] (tp = terephthalic acid and pyz = pyrazine) was synthesized through a hydrothermal method with metal centers equally occupied by Co^2+^ and Ni^2+^ ions [15]. The Ni/Co–MOF exhibited an outstanding specific capacitance of 1049 F·g^−1^ at a discharge current density of 1 A·g^−1^ in a 3 mol·L^−1^ KOH electrolyte. The Ni/Co-MOF synthesized via hydrothermal BTC route with a dandelion-like hollow structure shows an excellent specific capacitance of 758 F·g^−1^ at 1 A·g^−1^ in the three-electrode system [16]. Rahmanifar et al. [17] adopted a one-pot refluxing method to synthesize Ni/Co-MOF-rGO nanocomposite, which nanocomposite demonstrates a high specific capacitance of 860 F·g^−1^ at 1.0 A·g^−1^. The asymmetric activated carbon//Ni/Co-MOF-rGO device delivers specific energy of 72.8 W·kg^−1^ at 850 W·kg^−1^ and still holds 15.1 W·kg^−1^ under the high specific power of 42.5 kW·kg^−1^, as well as a long cycle life (91.6% capacitance retention after 6000 charge-discharge cycles at 1 A·g^−1^).

The objective of this work is to construct a three-dimensional (3D) hierarchical nanostructured structure that takes advantage of BMOF with a regular shape and submicron size without additional conductive carbon. Herein, we fabricated a NiCo-bimetal–organic framework (NCMF) with a 3D nanorod-like structure using a facile solvo-hydrothermal method using 1,3,5-benzenetricarboxylic acid (C_9_H_6_O_6_ or trimesic acid, BTC) as an organic compound. Furthermore, in the current study, single metal MOFs (Ni-MOF) with similar morphologies to that of the bimetal–organic framework were prepared for comparison. Structural and morphological properties of as-prepared NCMF are characterized by the following different techniques: X-ray diffraction (XRD), X-ray photoelectron spectroscopy (XPS), field-emission scanning electron microscopy (FE-SEM), energy dispersive X-ray spectroscopy (EDS), and Brunauer–Emmett–Teller (BET) analysis. The NCMF is investigated as the positive electrode in an NCMF//AC asymmetric supercapacitor device with gel electrolyte. The specific capacitance of the NCMFs was estimated in coin cell devices and three-electrode systems. A maximum specific capacitance of 1243 F·g^−1^ was achieved at 0.5 A·g^−1^ for the NCMF electrode. This novel 3D hierarchical nanorod-like NCMF electrode system could be interesting for the fabrication of high-performance supercapacitors under gel electrolyte media.

## 2. Materials and Methods

### 2.1. Materials

Nickel(II) nitrate hexahydrate (Ni(NO_3_)_2_·6H_2_O), urea (CH_4_N_2_O), deionized water, ethanol (C_2_H_5_OH), and 20% hydrochloric acid solution were purchased from Daejung Co. Ltd. (Sasang-gu, Busan, South Korea). Cobalt nitrate hexahydrate(Co(NO_3_)_2_·6H_2_O) was purchased from Junsei Chemical Co. Ltd. (Tokyo, Japan) Trimesic acid and polyvinylpyrrolidone (PVP) *M*_w_ = 40,000 g·mol^−1^) were purchased from Sigma-Aldrich Co. Ltd. (Saint-Louis, MI, USA) All commercial chemicals were used without any further purification.

### 2.2. Synthesis of Ni-MOF (i.e., NMF) or Co-MOF (i.e., CMF) or NCMF

**Figure 1:**
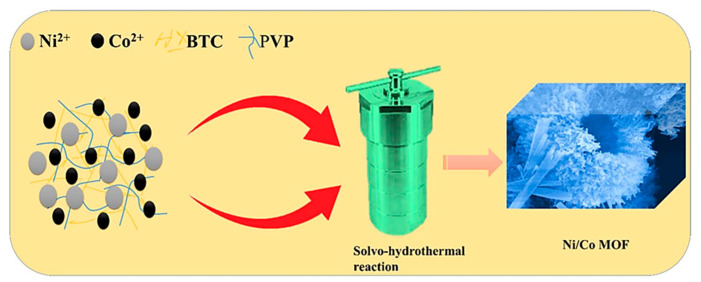
Schematic diagram of the Ni/Co MOF synthesis process.

Figure 1 shows the synthetic procedure for the preparation of NCMF. Mixture of anhydrous ethanol, de-ionized (DI) water, and dimethylformamide (DMF) in the ratio of 1:1:1 was stirred for 10 min to make solvent precursor. Add 411 mg of nickel nitrate hexahydrate (Ni(NO_3_)_2_·6H_2_O), cobalt nitrate hexahydrate (Co(NO_3_)_2_·6H_2_O), and 1200 mg of PVP to 80 mL of the solvent mixture. Add 615 mg of trimesic acid (BTC) to the remaining 130 mL of the solvent mixture. After stirring for 10 min to dissolve the material, dropwise mixing of the BTC solution into the above resultant solutions. After mixing well to obtain the precursor solution, pour the precursor solution into the autoclave reactor kept at 170 °C for 6 h. The obtained precipitate was washed twice by centrifugation with pure water and anhydrous ethanol, respectively, and dried in a vacuum oven at 80 °C for 10 h to finally obtain NCMF. For NMF and CMF preparation, the same procedure was used to replace the nickel precursor with cobalt nitrate hexahydrate (Co(NO_3_)_2_·6H_2_O). For comparison, an NCMF specimen was prepared without PVP during synthesis, denoted as NCMF-NP. Photographs of Ni-MOF, Co-MOF, and NiCo-MOF as-prepared powders are displayed in Figure S1. After doping of Co ions, the light green color of NMF changes to brown, which reveals that Ni ions have been substituted by Co ions in NCMF specimen.

### 2.3. Pre-Treatment of Ni-Foam

Foam nickel with a thickness of 2 mm was used as substrate. Before cleaning, the nickel foam was cut into rectangles of 15 × 5 mm^2^. Then immerse the nickel foam in a solution of 20% hydrochloric acid solution and deionized water mixed in a ratio of 3:1 and wash in an ultrasonic cleaner for 14 min to remove the surface oxide. After that, soak the nickel foam in deionized water and clean it in the ultrasonic cleaner for 14 min replace with new deionized water when finished, ultrasonic clean for 1 min, and repeat six times. Finally, replace the deionized water with ethanol and clean with the same procedure to remove the residual acid and organic matter from the surface. Dry in a vacuum oven at 90 °C for 12 h.

### 2.4. Characterizations

The structural features of as-prepared materials were analyzed by powder X-ray diffraction (X-ray diffractometer model XRD-6100, Shimadzu, Kyoto, Japan) with CuKα X-ray radiation (λ = 0.15406 nm). The morphological features were examined by scanning electron microscopy (FESEM, Hitachi, S-4800 and HRTEM, Tecnai G2 F20 S-Twin at an accelerating voltage of 200 kV). The elements of active materials were recognized using energy-dispersive X-ray spectroscopy (EDS) attached to the SEM. Sample mappings were obtained using annular dark-field imaging in a scanning transmission electron microscope (STEM) equipped with a high-angle annular dark field (HAADF) detector. The chemical states of the materials were tested using a Thermo Scientific X-ray photoelectron spectroscopy (XPS) instrument utilizing Al Kα radiation (λ = 1486.6 eV). The Brunauer–Emmett–Teller (BET) specific surface area was examined by N_2_ adsorption-desorption measurements in a Micromeritics ASAP 2420 surface area analyzer. The samples were evacuated at 150 °C before the N_2_ adsorption test. The BET surface area was estimated by the multipoint BET method based on the adsorption data in the *P/P*_0_ range of 0.0–1.0, where *P* and *P*_0_ correspond to the equilibrium and saturation pressures of the adsorbates at the temperature of adsorption, respectively.

### 2.5. Electrochemical Tests

The electrochemical activity of the electrodes was tested using a standard three-electrode cell, which consists of Hg/HgO and platinum mesh as the reference electrode and counter electrode. The working electrode was organized by mixing the active material, carbon black, and polyvinylidene difluoride (PVDF) in a mass ratio of 8:1.5:0.5 with N-methyl-2-pyrrolidone (NMP). This obtained slurry was then covered on nickel foam via the drop-casting technique and dried in an oven at 90 °C for 12 h. Cyclic voltammetry (CV), galvanostatic charge-discharge (GCD), and electrochemical impedance spectroscopy (EIS) were used to assess the electrochemical activity of the electrodes. The CV tests were carried out at several scan rates, ranging from 5 to 300 mV·s^−1^ at a potential of 0.0 V to 0.7 V in a 1 mol·L^−1^ KOH aqueous solution. The GCD tests were executed within the range of 0–0.5 V vs. Hg/HgO at various current densities. The electrochemical impedance spectroscopy (EIS) measurements were carried out in the frequency range from 100 Hz to 1 MHz at the open-circuit potential. All electrochemical experiments were performed using a Biologic SP-200 electrochemical workstation.

### 2.6. Preparation of Gel Electrolyte

In order to prepare the alkaline polyvinyl alcohol/potassium hydroxide (PVA/KOH) gel electrolyte, initially 5.6 g of PVA was dissolved in 50 mL of pure deionized water at 90 °C the temperature with continuous vigorous stirring to obtain a clear solution. As a result, after 1 h we obtained a clear, viscous solution. In total, 6 g of KOH was liquefied in 10 mL deionized water, then dropped into the cleared PVA solution with continuous stirring until complete dissolution and formation of a gel-like solution; finally, PVA/KOH gel electrolyte was cooled to room temperature for further use.

### 2.7. Ni/Co-MOF//AC Device Fabrication

A hybrid pouch-cell-type asymmetric supercapacitor device (HPASDs) was developed with in-situ synthesized Ni/Co-MOF@NF nanostructure as positive electrode, and active carbon and PVDF with Nafion (5 μL) in a mass ratio of 95:5 slurry was drop cast on a nickel foam to act as the negative electrode, separated with filter paper as separator. The Ni/Co-MOF@NF nanostructure was estimated from weight change of the nickel foam before and after deposition. The specific capacitance (*C_s_*) from charge–discharge curves in a three-electrode cell was intended using Equation (1) as follows [10]:

(1) $$C_{s}=\frac{I∆t}{m∆V},$$

where *I* (mA) and *t* (s) are the discharge current and discharge time, Δ*V* (V) is the voltage drop upon discharging (apart from the *IR* drop), and *m* (mg) is the mass of the active material. In addition, the energy density (*E_d_*) (W·kg^−1^) and power density (*P_d_*) (W·kg^−1^) of the device were estimated on the total mass of the active materials, as per Equations (2) and (3) as follows:

(2) $$E_{d}=\frac{1}{2}\left[\frac{C_{s}{\left(V_{f}-V_{i}\right)}^{2}}{3.6}\right],$$

(3) $$P_{d}=\frac{3600\times E_{d}}{∆t},$$

where ∆t and ($V_{f}-V_{i})$ are discharge time (s) and potential window for discharge process (V), respectively.

## 3. Results and Discussion

### 3.1. Structural and Morphological Studies

**Figure 2:**
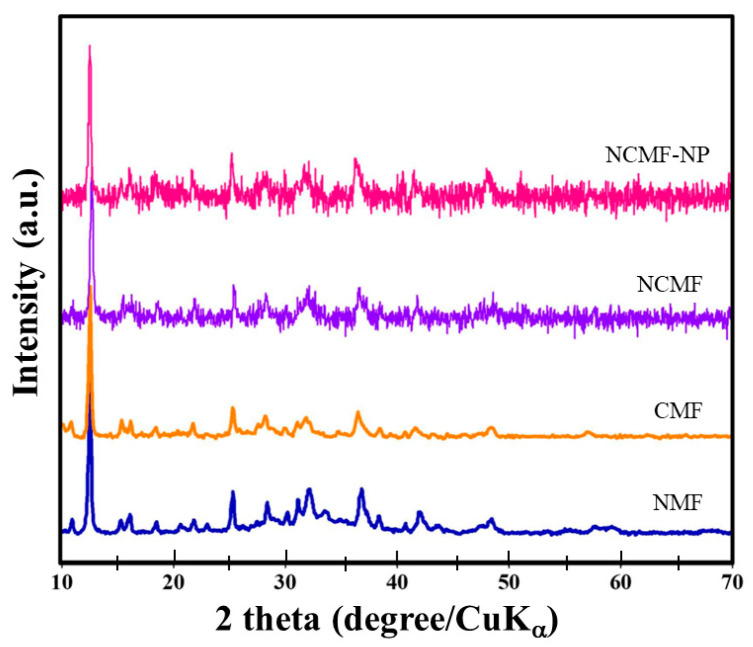
XRD patterns of the NMF, CMF, NCMF, and NCMF-NP specimens.

Figure 2 shows the XRD patterns of Ni-MOF (NMF), Co-MOF (CMF), NCMF, and NCMF-NP. The XRD patterns of NMF and CMF are very similar, and the sharpness of the respective peaks indicates crystallinity. For NMF, the diffraction peaks at approximately 12° are assigned to the (300) plane [18]. NCMF shows an XRD pattern alike to that of NMF, indicating that the crystal structure is not affected by the addition of the PVP, which is a non-ionic polymer with C=O, C-N, and CH_2_ functional groups that is widely used in nanoparticle synthesis. The contribution of PVP to obtaining nanostructured materials has been investigated by Kozkur et al. [19]. PVP can serve as a surface stabilizer, growth modifier, nanoparticle dispersant, and reducing agent. As shown with examples, its role depends on the synthetic conditions. This dependence arises from the amphiphilic nature of PVP along with the molecular weight of the selected PVP. Cao et al. [20] showed that PVP facilitated the subsequent nucleation and growth of MOF particles on their surfaces. Recently, Liu et al. [21] used PVP as a protective layer and dispersant for the synthesis of nanoparticles with concave cube morphology via a self-template eco-friendly method.

The XRD peaks of the NCMF sample are observed at 12.67°, 25.1°, and 36.8°, which is consistent with previous reports on NCMF [14]. The main peaks of NCMF at 12.67° and 25.1° correspond to *d*-spacings of 0.39 nm of the (110) plane and 0.19 nm of the (200) plane, respectively [22]. The XRD pattern for synthesized samples is identical to the reported data [14,22]. The position of the peak matched well with the simulated Ni-MOFs (Crystal Cambridge Data Center (CCDC) No. 1274034) [23]. The structure of Ni-MOF is composed of zigzag chains constructed from two symmetry-inequivalent tetra-aqua nickel(II) units and BTC ligands, as shown in Figure S2. In NCMF, the 2D layers are formed by the octahedral coordination of both Ni and Co atoms by six oxygen atoms from BTC, such that each 2D bimetal layer is separated by the linker molecules.

**Figure 3:**
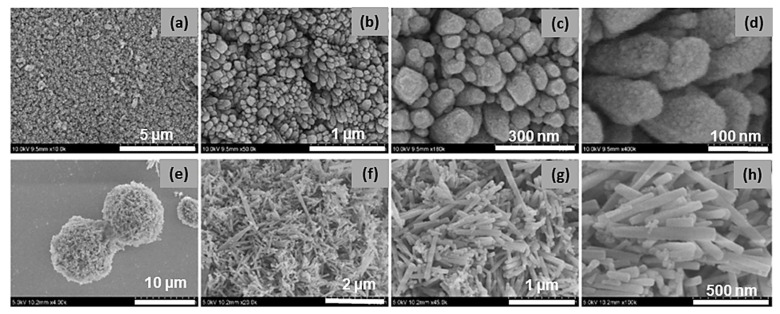
Different magnifications of FESEM images of (**a**–**d**) NMF and (**e**–**h**) NCMF-NP specimens.

**Figure 4:**
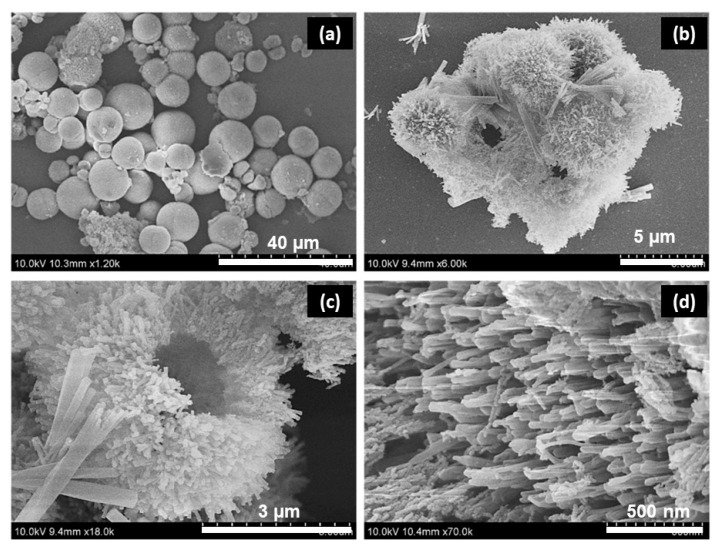
FESEM images of the NCMF specimen at different magnifications: (**a**) scale of 40 µm, (**b**) scale of 5 µm, (**c**) scale of 3 µm and (**d**) scale of 500 nm.

Figure 3 presents the FE-SEM images of NMF and NCMF-NP samples. Figure 3a–d shows that the tiny nanoparticles are well-arranged in a ribbon-like structure, while NCMF-NPs (Figure 3e–h) show randomly distributed multifaceted nanorod-like structures. Figure 4 displays the typical morphological features of NCMF, as analyzed via field-emission scanning electron microscopy. The morphology of the NCMF samples comprises aggregated and stacked microspheres with an average diameter of 1–3 μm, which consist of tightly connected bundles of extended nanorods, some of which are unfolded at their centers. These widely open distribution architectures favor the formation of large, exposed gaps with abundant exposed active sites, which can promote ion-inserted active centers through adequate contact with the electrolyte, thereby enhancing electrochemical activity.

**Figure 5:**
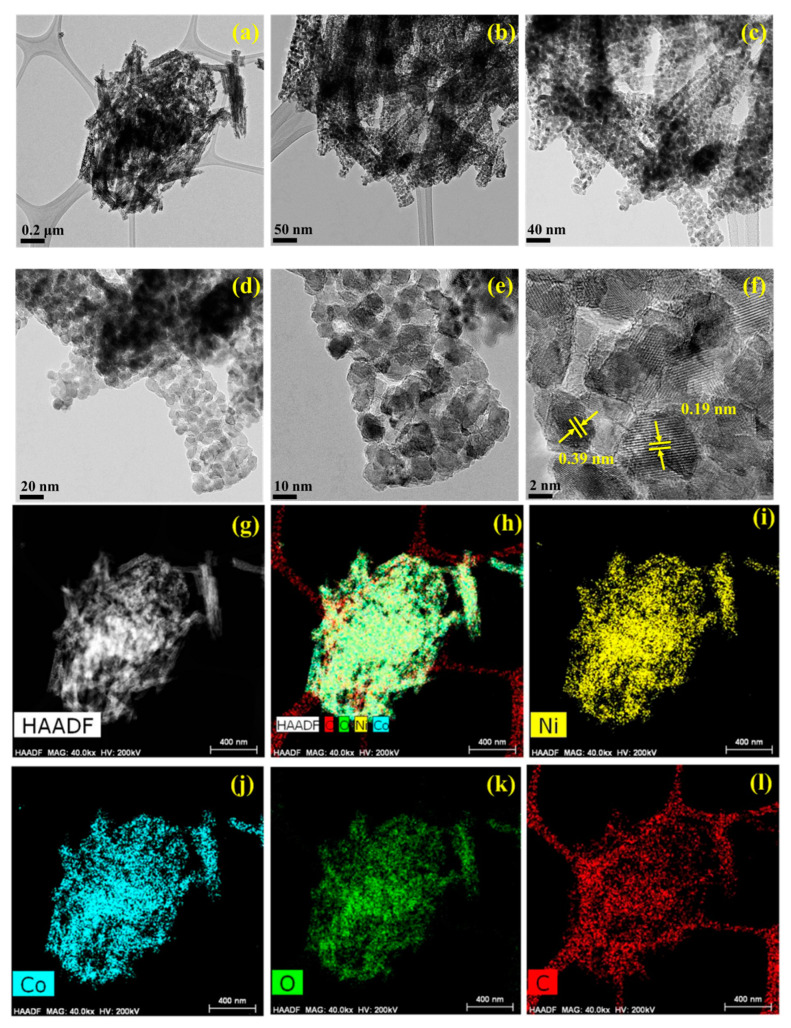
(**a**–**f**) HRTEM images of the NCMF specimen and (**g**–**l**) elemental mapping analyzed by the high-angle annular dark field (HAADF) technique.

The detailed morphological features of NCMF were investigated via high-resolution transmission electron microscopy (HRTEM) and elemental mapping. Figure 5 shows the typical HRTEM images of NCMF at different magnifications. These pictures evidence that a rod-like structure formed by Ni/Co NPs is tightly intertwined with dense and tiny NPs, which can enlarge the electrochemically active sites. The corresponding elemental maps analyzed by the high-angle annular dark field (HAADF) technique confirm the introduction of Ni, Co, and O species into NCMF. The Ni and Co plots are brighter than those of other elements, indicating higher content levels of Ni and Co. In Figure 5f, the *d*-spacings of 0.19 and 0.39 nm correspond to the (200) and (110) planes, respectively.

**Figure 6:**
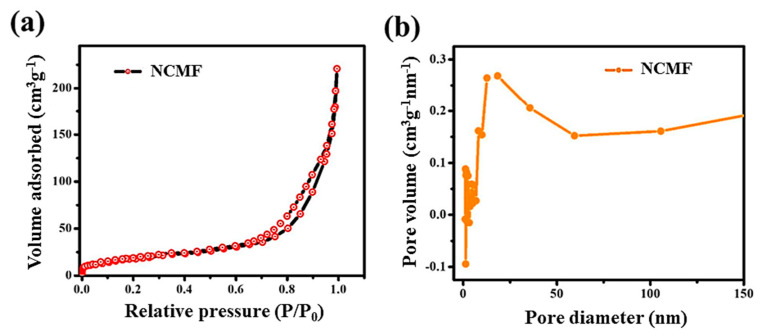
(**a**) N_2_ adsorption-desorption isotherm of NCMF and (**b**) pore size distribution determined by the BJH method.

Brunauer–Emmett–Teller (BET) analysis was carried out to estimate the specific surface area (SSA) and the mesoporous nature of the NCMF specimen from its nitrogen adsorption–desorption isotherms. The pore size distribution (PSD) was estimated by the Barrett–Joyner–Halenda (BJH) method. The N_2_ adsorption-desorption isotherm and pore size distribution of NCMF are shown in Figure 6a,b, respectively. The estimated BET SSA is 75 ± 5 m^2^·g^−1^. The standard isotherm of NCMF reveals that the MOFs contain both micropores and mesopores with maxima centered at 2 and 17 nm, respectively. The BET of NMF has already been documented, and we have considered and compared this source material. Li and co-workers reported a BET surface area of 67 m^2^·g^−1^ and an average pore size of 8.55 nm [24]. NCMF has a typical type IV isotherm with an H4-type hysteresis loop and a large apparent BET SSA, which is attributed to the hierarchical aggregation of NPs into rod-like structures and then into microspheres that may facilitate the exposure of electrochemically active sites [25]. Therefore, the large specific surface area of the NCMF is expected to afford good electrochemical performance. The obtained specific BET surface area for the NCMF compared with data in the literature are summarized in Table S1 [12,13,15,23,26,27,28,29]. These data show that the specific hydrothermal process assisted by PVP as a reducing agent provides a hierarchical inner pore structure favorable to high electrochemical activity.

### 3.2. XPS Studies

**Figure 7:**
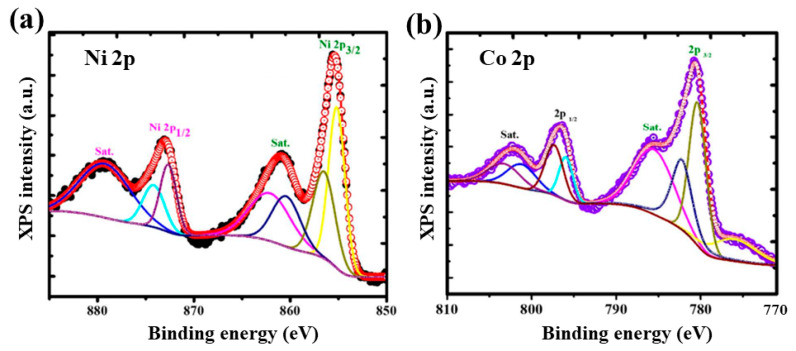
(**a**) XPS narrow scan spectra of (**a**) Ni 2p and (**b**) Co 2p of NCMF specimen.

The survey scan spectrum of NCMF in the binding energy range 0–1000 eV, demonstrating the main component signals (Ni, Co, and O), is shown in Figure S3. In the high-resolution Ni 2p spectrum of NCMF (Figure 7a), the two major peaks at 855.47 and 873.02 eV (peak separation Δ*E*_b_ = 17.55 eV) are ascribed to the Ni^2+^ 2p_3/2_ and Ni^2+^ 2p_1/2_, respectively. Additionally, the corresponding satellite peaks are detected at 860.94 and 879.43 eV (with Δ*E*_b_ = 18.49 eV), respectively. The results indicate that Ni exists in the divalent state [30]. Figure 7b shows the Co 2p XPS spectrum of NCMF; the two major peaks at 780.72 and 796.51 eV (with a binding energy difference Δ*E*_b_ = 15.79 eV) are attributed to Co 2p_3/2_ and Co 2p_1/2_, with satellite peaks at 785.76 and 802.45 eV (with Δ*E*_b_ = 16.69 eV), respectively; these observations confirm the presence of Co^2+^ in NCMF [31].

### 3.3. Electrochemical Studies—Three Electrode System

**Figure 8:**
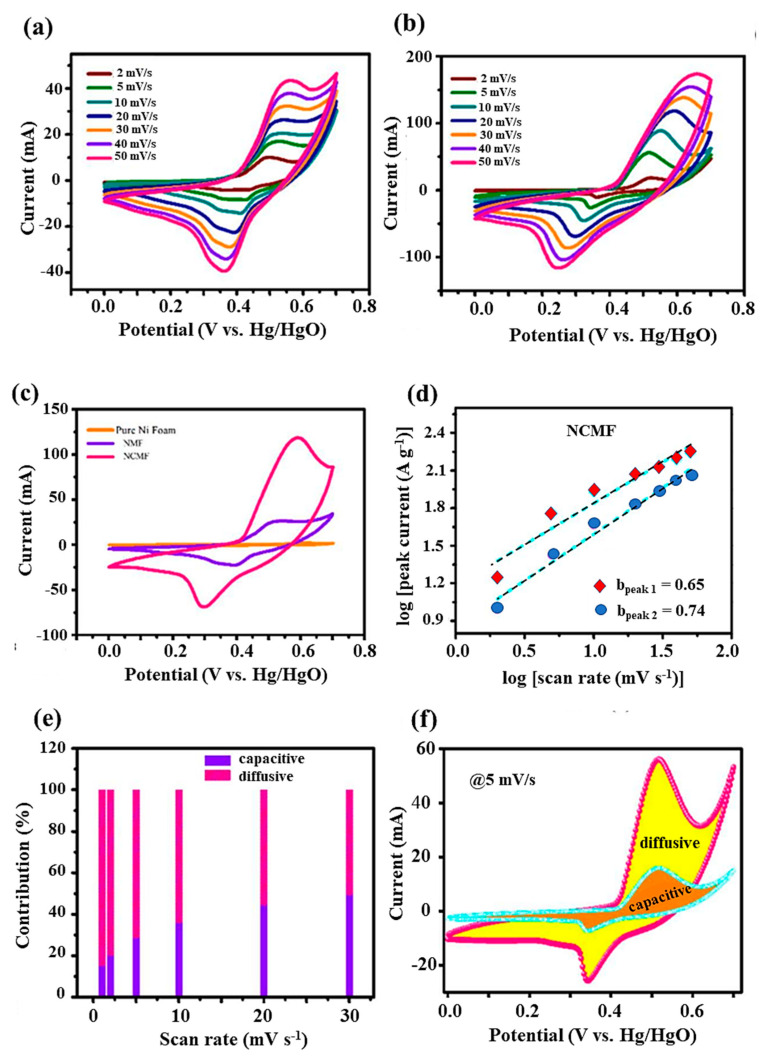
Three-electrode performance: CV curves of (**a**) NMF, (**b**) NCMF, (**c**) comparison of CV curves of bare nickel foam, NMF, and NCMF at 20 mV·s^−1^, (**d**) analysis of b value of the cathodic and anodic peaks of NCMF at different scan rates, (**e**) capacitive and diffusion contribution of NCMF, and (**f**) capacitive and diffusive contribution of NCMF electrode at 5 mV·s^−1^.

Cyclic voltammetry (CV) and galvanostatic charge-discharge (GCD) measurements have been performed to evaluate the specific capacitance, energy density, and power density of the fabricated electrodes. The electrochemical performances of NMF and NCMF electrodes were initially examined via cyclic voltammetry (CV) using the standard three-electrode configuration in a 1 mol·L^−1^ KOH electrolyte solution. The CV responses were recorded at various scan rates (2–50 mV·s^−1^) within the potential range of −0.1–0.8 V vs. Hg/HgO (Figure 8a,b). The voltammograms clearly display the distinct redox peaks during the anodic and cathodic sweeps and their contribution to the Faradaic pseudocapacitance. With the increase of scan rate from 2 to 50 mV·s^−1^, the redox peak separation Δ*E*_redox_ increased from 160 to 400 mV because of the electrode overpotential. Even at a high scan rate of 50 mV·s^−1^, the redox peaks are still significantly resolved, indicating the good rate capability of the NCMF electrode material. 

The NCMF electrode exhibits a higher current density than the NMF electrode, which is attributed to the redox properties of Ni and Co. To demonstrate the supercapacitive performance of the NCMF, the CV curves of NF, NMF, and NCMF electrodes are compared in the potential window of 0.7 V at 20 mV·s^−1^ scan rate (Figure 8c). The current response of nickel foam is insignificant, whereas the NCMF electrode shows a larger CV curve area than that of NMF, indicating that the NCMF electrode material is more electrochemically active and has considerable specific capacitance. The larger CV curve area of NCMF is attributed to the greater number of redox sites and exposure of these active sites, thus enhancing the electrochemical activity.

In order to distinguish the relative charge storage contribution from diffusion-controlled and surface effects, the general approach for the analysis of the peak current *i*_p_ can be described by a power law *i*_p_ = *K*ν^b^, where ν is the scanning rate and *K* and *b* are arbitrary coefficients. The coefficient *b* can vary from 0.5 to 1.0, with *b* = 0.5 being characteristic of a pure diffusion-limited process (charge storage via ion insertion) and *b* = 1.0 being characteristic of a capacitance process (charge storage via surface capacitance effects). From the slope of log *i*_p_ vs. log ν (Figure 8d), the calculated *b* values of the anodic peaks for the NMF and NCMF electrode materials are 0.74 and 0.65, respectively, indicating that the simultaneous pseudocapacitive response is the result of a combination of capacitive an insertion (Faradaic) process. It is worth noting that the electrochemical behavior of the NCMF electrode displays a more dominant Faradaic contribution because of the presence of cobalt sites in its framework. The capacitive contributions of NCMF at scan rates of 1, 2, 5, 10, 20, and 30 mV·s^−1^ are 15.08, 20.07, 28.42, 35.96, 44.27, and 49.31%, indicating a capacitive effect on the total capacitance with respect to the sweep rate (Figure 8e). The capacitive contribution increases with the scan rate owing to the increase in the ion transport motion and shortening of the diffusion pathways. Figure 8f shows the diffusive and capacitive contributions of the CV curve at 5 mV·s^−1^, revealing that the diffusive contribution is predominantly associated with the NCMF electrode, owing to the dual effect of Ni and Co ions on the electrochemical performance.

**Figure 9:**
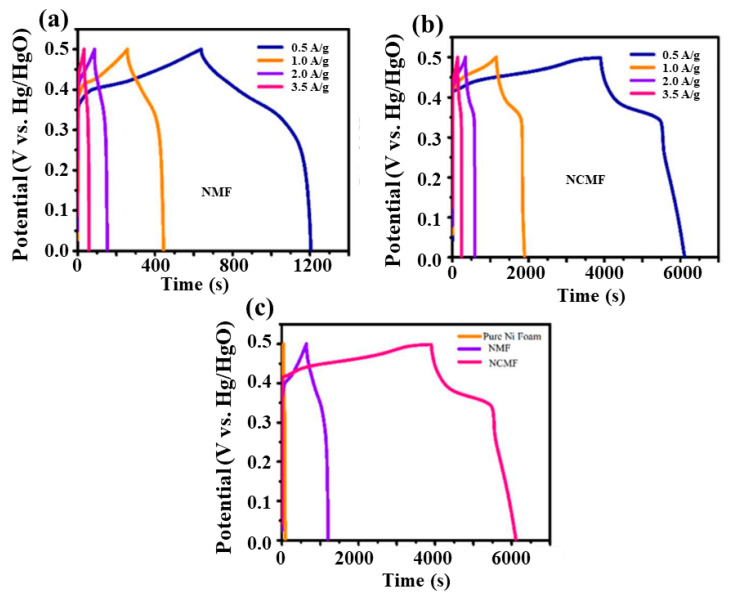
Charge/discharge curves at different current densities of NMF (**a**) and NCMF (**b**). Comparison of charge discharge curves of bare nickel foam, NMF, and NCMF electrodes (**c**).

Figure 9a,b present the galvanostatic charge–discharge (GCD) curves of NMF and NCMF electrodes at various current densities in the range 0.5–3.5 A·g^−1^. Figure 9c compares the GCD curves for NF, NMF, and NCMF electrodes recorded at 0.5 A·g^−1^. The NCMF electrode exhibits a longer discharge time than the NMF electrode, indicating a larger capacitance of the former. The recorded potential profiles of both electrodes show nonlinear GCD patterns that are comparable to the redox properties and indicate a battery-like behavior, i.e., the occurrence of quasi-reversible Faradaic reactions. Because of these Faradaic reactions, the charge/voltage ratio does not remain constant and varies with time. The longest discharge time of the NCMF electrode is attributed to the redox properties of Ni and Co ions, which is in good agreement with the CV curves. 

The porous NCMF electrode exhibits a faster ion transfer rate to the interior, which enhances its electrochemical performance with a rapid *I−V* response. Based on the discharge curves, the specific capacitance (*C_s_*) of the NCMF electrode material at different constant discharge currents can be calculated according to the expression given in Equation (1).

**Figure 10:**
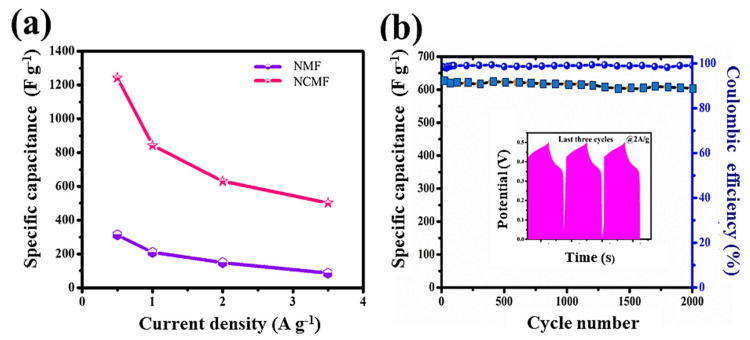
(**a**) Specific capacitance versus current density of NMF and NCMF electrodes and (**b**) Specific capacitance of NCMF electrode (squares) and Coulombic efficiency (bullets) as a function of cycle number (inset: last three cycles of GCD curves).

Figure 10a shows the specific capacitance with respective current densities for both NMF and NCMF electrodes. The specific capacitance of the NCMF electrode is 1243, 842, 631, and 501 F·g^−1^ at 0.5, 1.0, 2.0, and 3.5 A·g^−1^, respectively. At the same current densities, the NMF electrode exhibits specific capacitance values of 313, 209, 149, and 86 F·g^−1^, respectively. Therefore, the estimated specific capacitance of the NCMF electrode is approximately 4 times that of the NMF electrode, which is attributed to the synergetic interaction between Ni and Co ions, demonstrating its better rate capability facilitating faster electron transport and more efficient diffusion of ions into the redox sites of the composite material. The above comparative study also confirmed that the NCMF electrode would yield much higher current values. A comparison of the electrochemical performance of supercapacitors with various concentrations of KOH electrolyte is given in Table S2 [15,17,24,32,33,34,35]. Data show that the type and acid-base of the electrolyte solution have an impact on the electrochemical performance of the material tested in the three-electrode system.

Considering that the electrode life span is a crucial parameter of electrochemical devices, the cycling stabilities of the electrodes were assessed. Figure 10b displays the capacitance cyclability and the Coulombic efficiency (CE) of the NCMF electrode over 2000 cycles. The good long-term cycling stability of this electrode is confirmed as its capacitance retention is retained at 88.8% after 2000 cycles at 2 A·g^−1^. The electrochemical reversibility is evidenced by the last three GCD profiles exhibited in the inset. The NCMF electrode exhibits a CE of 98.22% at the first cycle and demonstrates a CE of 99.03% after 2000 cycles.

The electrochemical impedance spectroscopy (EIS) measurements were carried out over the frequency range from 100 Hz to 1 MHz at the open-circuit potential. The Nyquist plots −Z″(ω) vs. Z′(ω) of the NMF and NCMF electrodes are shown in Figure S4. The impedance spectra were fitted with an analogous circuit model (Figure S5). These graphs are composed of a depressed semicircle at the high-frequency region and a steeper line in the low-frequency range. The depressed semicircle corresponds to the charge transfer resistance (*R*_ct_) caused by Faradaic reactions. The steeper line evidences the capacitive nature of the electrode (it should be a vertical line for an ideal capacitor). The ohmic resistance (*R*_s_) of the electrode is calculated from the intercept of the real axis at the high-frequency range. *R*_s_ is the sum of the intrinsic resistance of electrode materials, the bulk resistance of the electrolyte, and the contact resistance at active materials/electrolyte/current collector interfaces. For both systems, *R*_s_ is 1.5 Ω. The calculated charge transfer resistances of the NMF and NCMF electrodes are 18.0 and 8.9 Ω, respectively, indicating an excellent charge transfer rate. Table S3 compares the electrochemical activities of NMF and NCMF electrodes with other popular MOF-based electrodes in previous reports, indicating that NCMF electrodes exhibit remarkable electrochemical activity [24,27,33,34,35,36,37,38.^39^,^40^,^41^,^42^,^43^,^44^,^45^,^46^].

### 3.4. Hybrid Pouch-Type Asymmetric Supercapacitor Device (HPASD)

**Figure 11:**
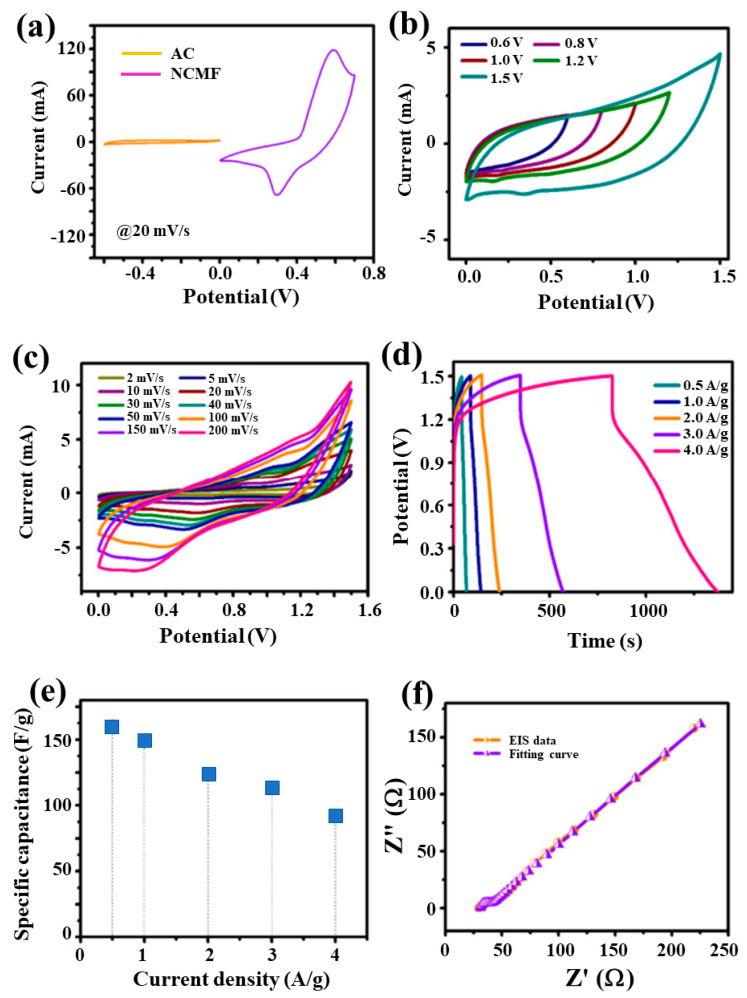
Electrochemical performance of the NCMF//AC device: (**a**) Comparison of the CV curves of AC and NCMF using three-electrode configuration at 20 mV·s^−1^, (**b**) CV curves of HPASD at different potentials; (**c**) CV profiles at different scan rates with a potential window of 1.5 V, (**d**) GCD profiles with different current densities, (**e**) specific capacitance vs. current densities, and (**f**) Nyquist plot with fitting curves of HPASD.

Based on the high electrochemical activity of the NCMF electrode, an NCMF//AC HPASD with the alkaline PVA/KOH gel electrolyte was fabricated and characterized. Figure 11a shows the CV curves of positive and negative electrodes recorded at a scanning rate of 20 mV·s^−1^, i.e., the NCMF three-electrode configuration in the potential range 0–0.7 V and the AC electrode in the potential range from −0.6 to 0 V. Figure 11b exhibits the CV profiles of the NCMF//AC HPASD at different applied potentials (0.6–1.5 V), implying that 0–1.5 V is the optimal potential window for HPASD. Figure 11c displays the CV profiles obtained at an optimized working potential of 1.5 V at different scan rates in the range of 2–200 mV·s^−1^. A steady increase in the CV curve area with scan rate indicates a good electrochemical behavior of the asymmetric device. Additionally, the charge–discharge curves of HPASD (Figure 11d) at different current densities in the range of 0.5–4 A·g^−1^ suggest a good rate capability. Figure 11e exhibits the plot of specific capacitance vs. current density for the HPASD. The estimated specific capacitances of the HPASD are 161, 147, 124, 112, and 91 F·g^−1^ at 0.5, 1, 2, 3, and 4 A·g^−1^, respectively. Note that the specific capacitance decreases almost linearly with the increase of current density at the rate of 19.3 F A^−1^. Figure 11f shows the EIS pattern of the HPASD along with the fitted curve. The estimated *R*_s_ and *R*_ct_ values for HPASD are 30.93 and 7.49 Ω, respectively. The straight line with a slope of 45◦ in the low-frequency region expresses the Warburg diffusion impedance.

**Figure 12:**
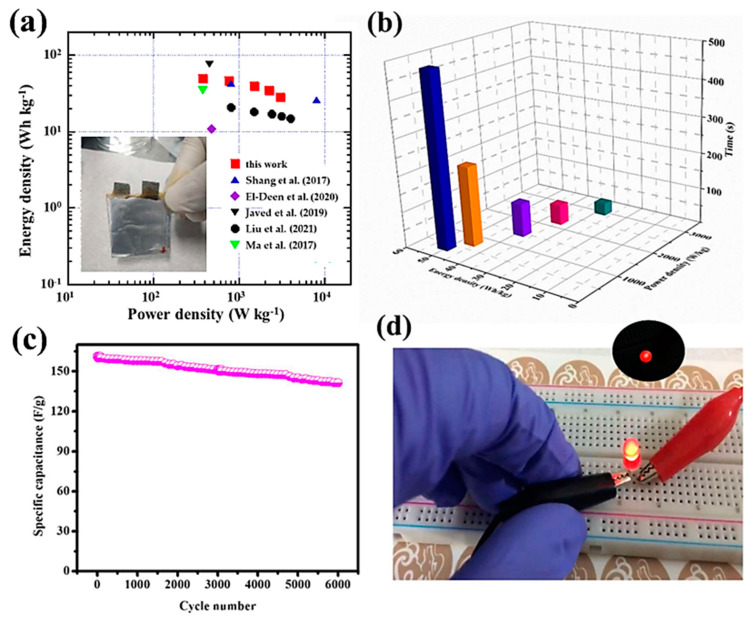
(**a**) Ragone plot (inset shows a laboratory prototype of the fabricated device) compared with data in the literature (Ma et al. [53], Shang et al. [54], El-Deen et al. [55], Javed et al. [56], Liu et al. [57]), (**b**) 3D plot of the electrochemical performance of the asymmetric supercapacitor prototype (energy density vs. power density vs. discharge time, (**c**) stability of the HPASD tested at 0.5 A·g^−1^ current density, and (**d**) Practical applications of the AC//Ni/Co-MOF device: digital images of a red-LED lit by the HPASD.

Energy and power densities are key parameters for the validation of the electrochemical performance of a hybrid asymmetric supercapacitor. The Ragone plot (Figure 12a) of HPASD, derived from the GCD curves based on Equations (2) and (3), shows that the HPASD delivers an energy density (*E_d_*) of 50.3 W·kg^−1^ at a power density (*P_d_*) of 375 W·kg^−1^. A comparison of energy and power densities of hybrid pouch-type asymmetric supercapacitor devices reported in the literature is provided in Table 1 [47,48,49,50,51,52,53,54,55,56,57,58,59,60,61,62,63,64]. The electrochemical characteristics of the as-fabricated HPASD are superior to the reported values in the literature [17,61,62,63]. Particularly, Tao et al. [61] demonstrated an *E_d_* of 36 W·kg^−1^ at a *P_d_* of 852 W·kg^−1^ combining the merits of MOF derivatives and the free-standing core–shell heterostructure leaf-like Co_3_O_4_@NiCo_2_O_4_ nanoarray electrode. Ye et al. [45] assembled a Ni–Co MOF//AC device with polybenzimidazole (PBI)/KOH as a solid electrolyte, which achieved a high specific capacitance of 172.7 F·g^−1^ at 0.5 A·g^−1^ in a large potential window of 1.8 V. The corresponding Ragone values, i.e., *E_d_* of 77.7 W·kg^−1^ and *P_d_* of 0.45 kW·kg^−1^, are close to that of our HPASD, but the construction implying solid-state interfaces is trickier than the PVA/KOH gel technology. Figure 12b shows a 3D plot of the energy vs. power density vs. discharge time. The significant energy-power densities and their long-term stability are crucial parameters for device applications. Figure 12c shows the stability–durability test results for the HPASD over 6000 charge-discharge cycles at a current density of 0.5 A·g^−1^. In this test, the NCMF//AC HPASD retained 87.6% of its initial capacitance, indicating a very high level of stability. To demonstrate the excellent charge storage properties of the developed asymmetric supercapacitor device, two HPASDs were connected in series, powering a red light-emitting diode (LED) (Figure 12d), and were able to power it for 70 s. This test suggests the potential applications of such NCMF//AC supercapacitors for wearable electronics.

## 4. Conclusions

In summary, a bimetallic Ni/Co metal–organic framework has been developed as electrode material for asymmetric supercapacitor devices. The Ni/Co MOF was successfully synthesized using a simple solvo-hydrothermal synthesis. The composition-tuned and morphology-controlled of the nanostructured Ni-MOF are important issues to enhance the electrochemical performance of supercapacitors. In the synthesis process, PVP has served as a surface stabilizer, growth modifier, nanoparticle dispersant, and reducing agent. As shown with examples, its role depends on the synthetic conditions. This dependence arises from the amphiphilic nature of PVP along with the molecular weight of the selected PVP. The coordinatively unsaturated transition-metal centers (Ni^2+^ and Co^2+^) are the dominant active sites for electrochemical reactions. The comparison of NMF and NCMF evidence that the incorporation of Co metal ions in the NMF matrix (Ni:Co = 50:50) greatly improves the capacitive properties.

As-prepared NCMF exhibited a 3D hierarchical nanorod structure with a high specific surface area and exhibited a maximum capacitance of 1243 F·g^−1^ at 0.5 A·g^−1^ and good cycling stability (88.8% after 4000 cycles). These results were attributed to the pronounced redox effect of Ni and Co species and to desirable morphological properties (rod-like structure, high specific surface area, well-defined pores, and good mesoporous characteristics). Additionally, NCMF was used without carbon additive as an electrode for HPASD (pouch-type assembly with AC as the negative electrode). The fabricated device exhibited high energy (50.3 W·kg^−1^) and power densities (375 W·kg^−1^). Furthermore, the proposed HPASD was able to power a red LED for 70 s. The energy density/power density ratio is the highest value reported so far after 6000 cycles, with a cycling stability of 87.6%. We believe that the ion (electrolyte) and pore (bimetallic MOF material) size has been properly matched for a better redox process. The increased capacitance and cyclic stability of bimetallic MOF-based SCs are clearly demonstrated as compared to mono metal MOF-based SCs, which could be attributed to the effect of bimetal ions on the electronic properties and to the high intrinsic porosity and surface area of nanorod-like particles. These results suggest that bimetallic MOFs could function as promising and innovative electrode materials for asymmetric supercapacitors to power wearable electronics. In diverse applications and especially in energy storage, a bimetallic MOF is expected to behave markedly differently from MOF, and it should possess sufficient stability in electrochemical reactions to last longer.

## Figures and Tables

**Table 1:** Comparison of energy and power densities of hybrid pouch-type asymmetric supercapacitor devices previously reported in the literature.

HPASD	Energy Density (W·kg^−1^)	Power Density (W·kg^−1^)	Ref.
Ni/Co-TC//AC	37	801	[47]
ZnCo_2_O_4_-C//AC	49.5	700	[48]
NiCoP//graphene films	33	1301	[49]
ZnCo_2_O_4_–ZnWO_4_//AC	24	400	[50]
NiO–C–rGO//AC	36	749.1	[51]
ZnCo_2_O_4_–MnO_2_//AC	29.4	628	[52]
ZnCo_2_O_4_@NG//AC	24	500	[53]
ZnCo_2_O_4_//AC	30	399	[54]
NiCo_2_O_4_/CNFs//carbon fibers	39	1600	[55]
Zn/Co–O//NPC	118	1491	[56]
CoFe_2_O_4_/CNFs//AC	21.4	850	[57]
O–NiCoP@rGO//AC	21	775	[58]
CoFe_2_O_4_/MWCNTs//AC	27	319	[59]
Ni–Co–MOF//AC	55.7	1000	[12]
CoNi_2_O_3_/CFP//AC	27	1450	[60]
Ni–Co–MOF//AC	34.3	375	[28]
Co_3_O_4_/NiCo_2_O_4_//AC	36	852	[61]
NCMF//rGO	42.2	800	[62]
Ni-Co MOF//AC	12.8	372	[63]
Ni-Co MOF//AC	77.7	450	[64]
Ni/Co-MOF//AC	20.9	800	[16]
Ni/Co-MOF-rGO//AC	72.8	850	[17]
NCMF//AC	50.3	375	this work

## Data Availability

All data are provided in this article.

## Supplementary Materials

The following supporting information can be downloaded at: https://www.mdpi.com/article/10.3390/ma16062423/s1, Preparation of materials and fabrication of supercapacitors. Figure S1: Photographs of Ni-MOF, Co-MOF and NiCo-MOF as-prepared powders via solvo-hydrothermal synthesis. Figure S2: The suggested structure of the bimetal MOF framework with Ni as the metal centers surrounded by the organic linker. Table S1: Comparison of BET surface area and of NCMF with values reported in the literature. Figure S3: XPS survey scan of the NCMF nanostructure. Table S2. Comparison of electrochemical performance with previous reports. Figure S4: Nyquist plots of NMF and NCMF electrodes (inset shows the magnified image of Nyquist plots in the high-frequency range). Figure S5: Equivalent circuit used for Nyquist plot fitting. Table S3: Comparison of electrochemical activity of NCMF electrode and other popular MOF-based electrodes in previous reports.
