# Supplementary material for: Pouch-Type Asymmetric Supercapacitor Based on Nickel–Cobalt Metal–Organic Framework
Source: Materials (Basel). 2023 Mar 17;16(6):2423. doi: 10.3390/ma16062423 (PMC10052718; doi:10.3390/ma16062423)
Supplement: Supplementary file 1 [file materials-16-02423-s001.zip › materials-2235030-supplementary.pdf]

materials

Supplementary Information

Pouch-Type Asymmetric Supercapacitors Based on Nickel–Cobalt Metal–Organic Frameworks

Surya. V. Prabhakar Vattikuti [1], Nguyen To Hoai [2,3], Jie Zeng [1] and Rajavaram Ramaraghavulu [4], Nam Nguyen Dang [2,3], Jaesool Shim [1] and Christian M. Julien [5]

1 School of Mechanical Engineering, Yeungnam University, 280 Daehak-Ro, Gyeongsan, Gyeongbuk, 712-749, South Korea
2 Future Materials & Devices Lab., Institute of Fundamental and Applied Sciences, Duy Tan University, Ho Chi Minh City 700000, Vietnam
3 The Faculty of Environmental and Chemical Engineering, Duy Tan University, Danang 550000, Vietnam
4 Department of Physics, School of Applied Sciences, REVA University, Bangalore 560064, India
5 Institut de Minéralogie, de Physique des Matériaux et de Cosmochimie (IMPMC), Sorbonne Université, CNRS-UMR 7590, 4 place Jussieu, 75252 Paris, France

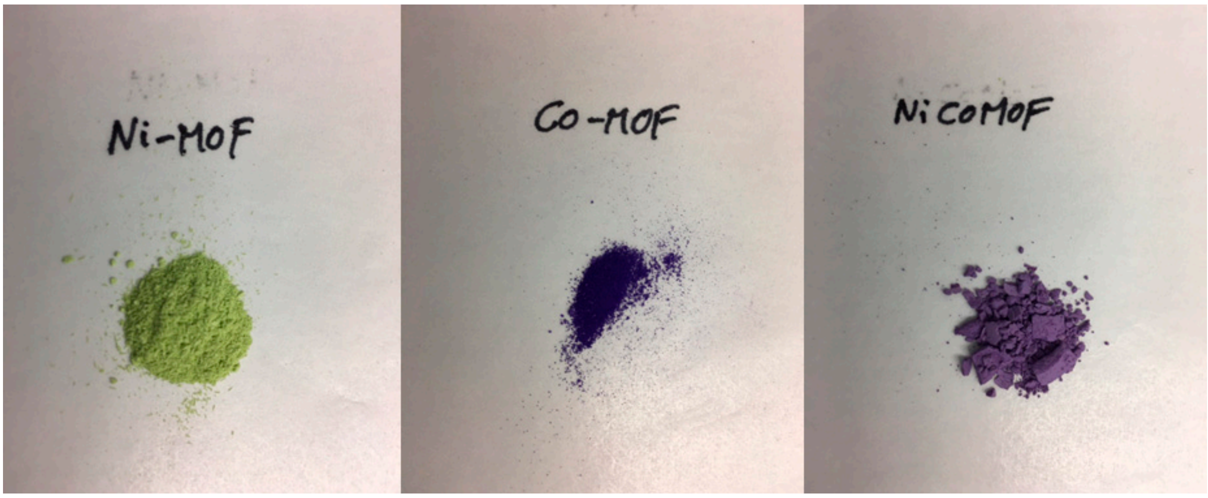

Figure S1. Photographs of Ni-MOF, Co-MOF and NiCo-MOF as-prepared powders via solvo-hydrothermal synthesis.

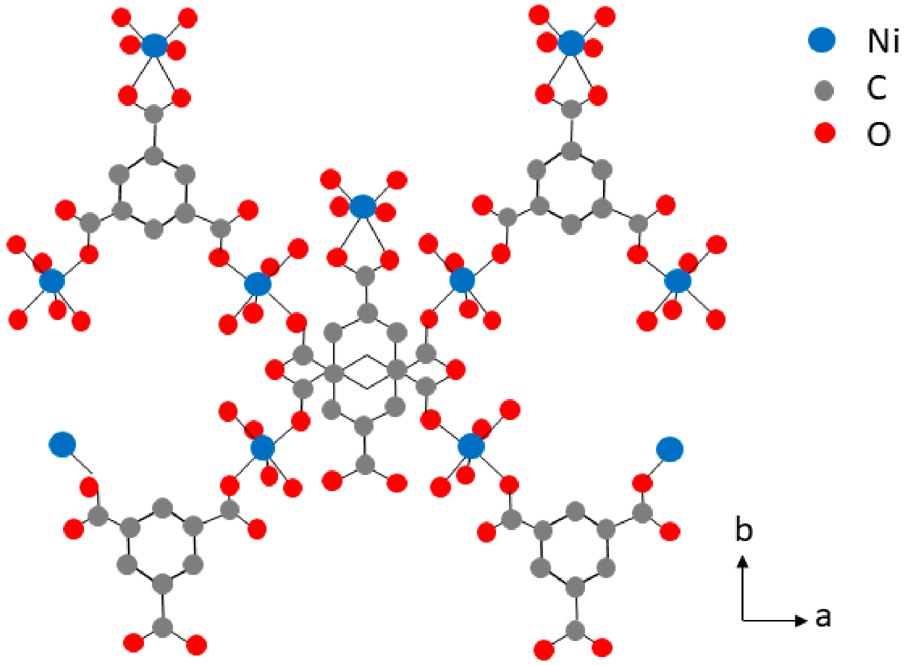

Figure S2. The suggested structure of the bimetal MOF framework with Ni as the metal centers surrounded by the organic linker.

Table S1. Comparison of BET surface area and of NCMF with values reported in the literature.

Material	Synthesis	SSA (m^2 g^{-1})	Average pore size (nm)	Ref.
Ni-Co(BDC)	Microwave	50.8	-	[1]
Ni-Co(BDC)	Hydrothermal	70.7	2-20	[2]
Ni(BTC)	Electrochemical	22.8	3-10	[3]
Ni-Co(BTC)	Hydrothermal	126.6	2.3	[4]
Co(BTC)	Hydrothermal	31.9	3	[5]
Ni-Co(tp+pyz)	Hydrothermal	775	0.85	[6]
Ni-Co(tp)	Hydrothermal	22	2.2	[7]
Ni(BTC)	Electrochemical	7.8	3.3	[8]
Ni-Co(BTC)	Hydrothermal	75.0	17	this work

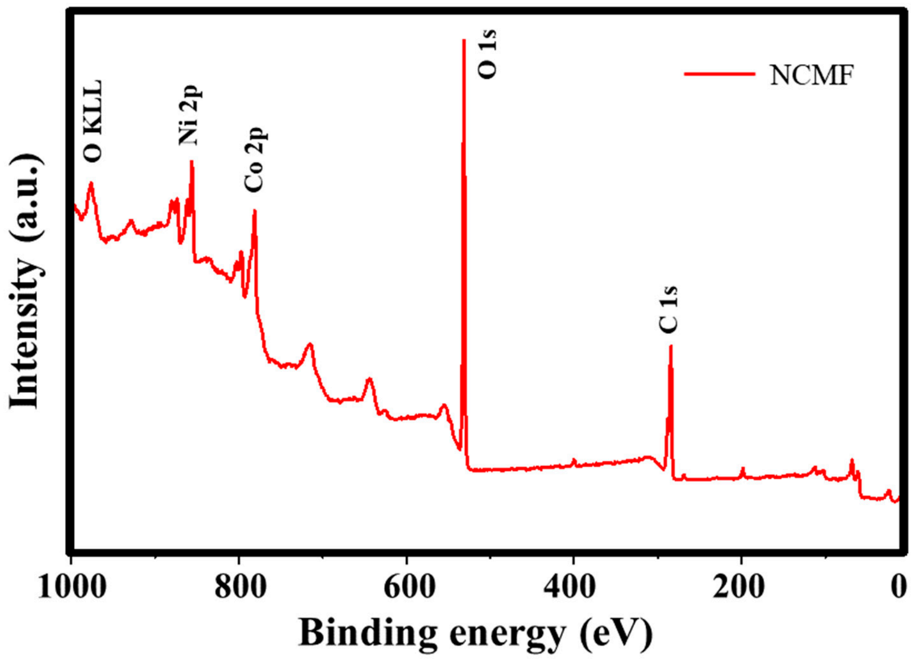

Figure S3. XPS survey scan of the NCMF nanostructure.

Table S2. Comparison of electrochemical performance with previous reports.

Material	Electrolyte	Performance C_s@rate	Ref.
Ni-Co-MOF (BTC)	3 M KOH	900 F g^{-1}@1 A g^{-1}	[9]
CoNi(μ_3-tp)$_2$(μ_2-pyz)$_2$	3 M KOH	1049 F g^{-1}@1 A g^{-1}	[10]
Co/Ni-MOF (BDC)	3 M KOH	236 mAh g^{-1}@1 A g^{-1}	[11]
Ni/Co-MOF-rGO (oba)	6 M KOH	860 F g^{-1}@1 A g^{-1}	[12]
Ni/Co-based MOF (BDC)	1 M KOH	827 C g^{-1}@20 mA cm^{-2}	[13]
Ni/Co-MOFs (BDC)	3 M KOH	1126 F g^{-1}@0.5 A g^{-1}	[14]
Ni-MOF (BTC)	6 M KOH	750 F g^{-1}@5 mV s^{-1}	[15]
Ni-Co-MOF (BTC)	1 M KOH	842 F g^{-1}@1 A g^{-1}	This work

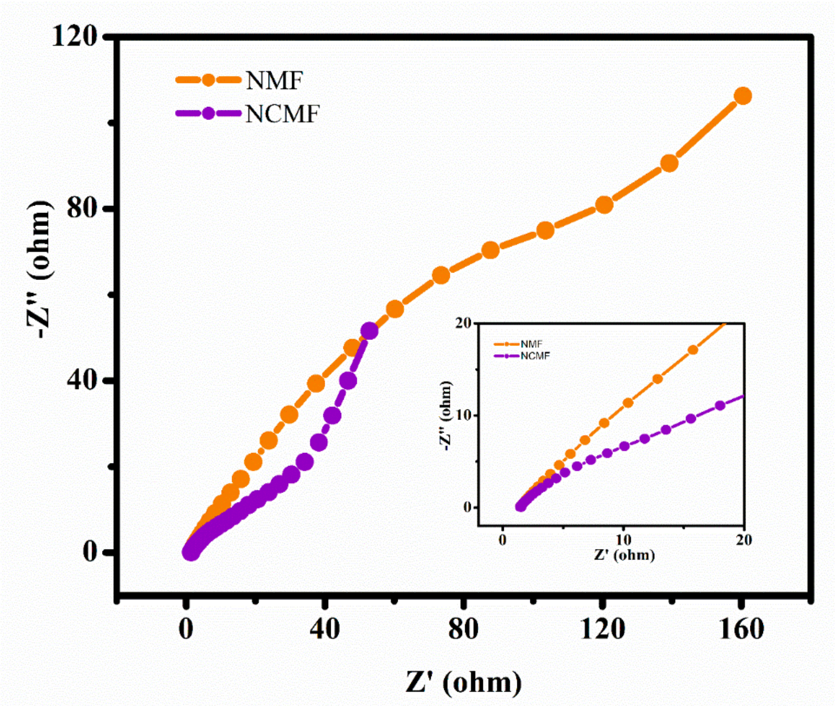

Figure S4. Nyquist plots of NMF and NCMF electrodes (inset shows the magnified image of Nyquist plots in the high-frequency range).

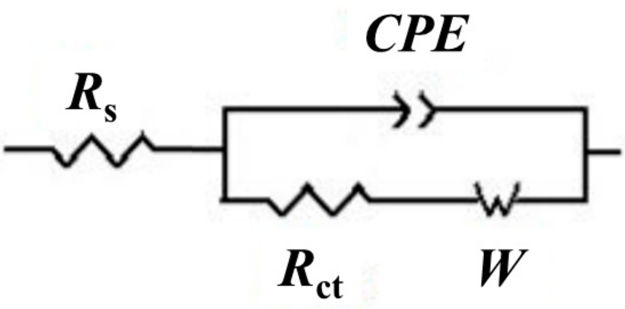

Figure S5. Randles equivalent circuit used for Nyquist plot fitting. *R*s represents the ohmic electrolyte resistance, R_{ct} the charge transfer resistance, *CPE* is the constant phase element replacing the ideal double-layer capacitor (C_{dl}) and *W* is the Warburg impedance.

Table S3. Comparison of electrochemical activity of NCMF electrode and other popular MOF-based electrodes in previous reports.

Materials	Electrolyte	Scan rate or current density	Specific capacitance (F g^{-1})	Ref.
Ni-CoMOF	3 M KOH	1 A g^{-1}	236.1 mAh g^{-1}	[16]
ZIF-67/polypyrrole nanotubes	1M Na_2SO_4	0.5 A g^{-1}	597.6	[17]
Ni@CoNi-MOF	6 M KOH	1 A cm^{-2}	772 C cm^{-2}	[18]
Ni-MOF-5/rGO	1 M KOH	1 mV s^{-1}	758	[19]
ZIF-8/PANI	1 M H_2SO_4	1 A g^{-1}	236	[20]
Cu MOF/rGO	PVA–Na_2SO_4	1 A g^{-1}	385	[21]
Ni MOF-derived NPs/graphene	1 M H_2SO_4	1 A g^{-1}	886	[22]
UIO-66/rGO	6 M KOH	0.15 A g^{-1}	302	[23]
PANI-ZIF-67-CC	3 M KCl	10 mV s^{-1}	21.5 mF cm^{-2}	[24]
ZIF-67/rGO composite	mixture[a)]	4.5 A g^{-1}	1453	[25]
Co-MOF	3 M KOH	2 A g^{-1}	958	[5]
Co-MOF	5 M KOH	1 A g^{-1}	2564	[26]
Ni-MOF/CNTs	3 M KOH	1 A g^{-1}	680 C g^{-1}	[27]
Ni-MOF	6 M KOH	5 mV s^{-1}	750	[15]
Ni-MOF	1 M KOH	0.5 A g^{-1}	414	this work
Ni/Co-MOF	1 M KOH	0.5 A g^{-1}	1213	this work

[a)]0.2 M $K_3[Fe(CN)_6]$ + 1 M Na_2SO_4

References

1. Kurisingal, J.F.; Babu, R.; Kim, S.-H.; Li, Y.X.; Chang, J.-S.; Cho, S.J.; Park, D.-W. Microwave-induced synthesis of a bimetallic charge-transfer metal organic framework: a promising host for the chemical fixation of CO_2. *Catal. Sci. Technol.* **2018**, *8*, 591-600.
2. Liang, X.; Quan, B.; Chen, J.; Tang, D.; Zhang, B.; Ji, G. Strong electric wave response derived from the hybrid of lotus roots-like composites with tunable permittivity. *Sci. Rep*. **2017**, *7*, 9462.
3. Cao, W.; Liu, Y.; Xu, F.; Li, J.; Li, D.; Du, G.; Chen, N. In situ electrochemical synthesis of rod-like Ni-MOFs as battery-type electrode for high performance hybrid supercapacitor. *J. Electrochem. Soc*. **2020**, *167*, 050503.
4. Radhika, M.G.; Gopalakrishna, B.; Chaitra, K.; Gopalakrishna Bhatta, L.K.; Venkatesh, K.; Sudha Kamath, M.K; Kathyayini, N. Electrochemical studies on Ni, Co & Ni/Co-MOFs for high-performance hybrid supercapacitors. *Mater. Res. Express* **2020**, *7*, 054003.
5. Ramachandran, R.; Zhao, C.; Luo, D.; Wang, K.; Wang, F. Morphology-dependent electrochemical properties of cobalt-based metal organic frameworks for supercapacitor electrode materials. *Electrochim. Acta* **2018**, *267*, 170-180.
6. Gholipour-Ranjbar, H.; Soleimani, M.; Naderi, H.R. Application of Ni/Co-based metal-organic frameworks (MOFs) as an advanced electrode material for supercapacitors. *New J. Chem.* **2016**, *40*, 9187-9193.
7. Wang, J.; Zhong, Q.; Zeng, Y.; Cheng, D.; Xiong, Y.; Bu, Y. Rational construction of triangle-like nickel-cobalt bimetallic metal-organic framework nanosheets arrays as battery-type electrodes for hybrid supercapacitors. *J. Colloid Interface Sci*. **2019**, *555*, 42-52.
8. Jabarian, S.; Ghaffarinejad, A. Electrochemical synthesis of NiBTC metal organic framework thin layer on nickel foam: An efficient electrocatalyst for the hydrogen evolution reaction. *J. Inorg. Organometal. Polymers Mater*. **2019**, *29*, 1565-1574.
9. Zhao, S.; Zeng, L.; Cheng, G.; Yu, L.; Zeng, H. Ni/Co-based metal-organic frameworks as electrode material for high performance supercapacitors. *Chinese Chem. Lett.* **2019**, *30*, 605-609.
10. Gholipour-Ranjbar, H.; Soleimani, M.; Naderi, H.R. Application of Ni/Co-based metal-organic frameworks (MOFs) as an advanced electrode material for supercapacitors. *New J. Chem.* **2016**, *40*, 9187-9193.
11. Jiao, Y.; Pei, J.; Chen, D.; Yan, C.; Hu, Y.; Zhang, Q.; Chen, G. Mixed-metallic MOF based electrode materials for high performance hybrid supercapacitors. *J. Mater. Chem. A* **2017**, *5*, 1094-1102.
12. Rahmanifar, M.S.; Hesari, H.; Noori, A.; Masoomi, M.Y.; Morsali, A.; Mousavi, M.F. A dual Ni/Co-MOF-reduced graphene oxide nanocomposite as a high performance supercapacitor electrode material. *Electrochim. Acta* **2018**, *275*, 76-86.
13. Xu, F.; Chen, N.; Fan, Z.; Du, G. Ni/Co-based metal organic frameworks rapidly synthesized in ambient environment for high energy and power hybrid supercapacitors. *Appl. Surf. Sci.* **2020**, *528*, 146920.

14. Sun, J.; Yu, X.; Zhao, S.; Chen, H.; Tao, K.; Han, L. Solvent-controlled morphology of amino-functionalized bimetal metal−organic frameworks for asymmetric supercapacitors. *Inorg. Chem.* **2020**, *59*, 11385-11395.
15. Li, X.; Li, J.; Zhang, Y.; Zhao, P.; Lei, R.; Yuan, B.; Xia, M. The evolution in electrochemical performance of honeycomb-like $Ni(OH)_2$ derived from MOF template with morphology as a high-performance electrode material for supercapacitors. *Materials* **2020**, *13*, 4870.
16. Jiao, Y.; Pei, J.; Chen, D.; Yan, C.; Hu, Y.; Zhang, Q.; Chen, G. Mixed-metallic MOF based electrode materials for high performance hybrid supercapacitors. *J. Mater. Chem. A* **2017**, *5*, 1094–1102.
17. Xu, X.; Tang, J.; Qian, H.; Hou, S.; Bando, Y.; Hossain, M.S.A.; Pan, L.; Yamauchi, Y. Three-dimensional networked metal-organic frameworks with conductive polypyrrole tubes for flexible supercapacitors. *ACS Appl. Mater. Interfaces* **2017**, *9*, 38737−38744.
18. Hong, M.; Zhou, C.; Xu, S.; Ye, X.; Yang, Z.; Zhang, L.; Zhou, Z.; Hu, N.; Zhang, Y. Bi-metal organic framework nanosheets assembled on nickel wire films for volumetric-energy-dense supercapacitors. *J. Power Sources* **2019**, *423*, 80–89.
19. Banerjee, P.C.; Lobo, D.E.; Middag, R.; Ng, W.K.; Shaibani, M.E.; Majumder, M. Electrochemical capacitance of Ni-doped metal organic framework and reduced graphene oxide composites: more than the sum of its parts. *ACS Appl. Mater. Interfaces* **2015**, *7*, 3655−3664.
20. Salunkhe, R.R.; Tang, J.; Kobayashi, N.; Kim, J.; Ide, Y.; Tominaka, S.; Kim, J.H.; Yamauchi, Y. Ultrahigh performance supercapacitors utilizing core-shell nanoarchitectures from a metalorganic framework-derived nanoporous carbon and a conducting polymer. *Chem. Sci.* **2016**, *7*, 5704−5713.
21. Srimuk, P.; Luanwuthi, S.; Krittayavathananon, A.; Sawangphruk, M. Solid-type supercapacitor of reduced graphene oxide-metal organic framework composite coated on carbon fiber paper. *Electrochim. Acta* **2015**, *157*, 69−77.
22. Wu, M.-S.; Hsu, W.-H. Nickel nanoparticles embedded in partially graphitic porous carbon fabricated by direct carbonization of nickel-organic framework for high-performance supercapacitors. J. *Power Sources* **2015**, *274*, 1055−1062.
23. Mao, M.L.; Sun, L.X.; Xu, F. Metal−organic frameworks/carboxyl graphene derived porous carbon as a promising supercapacitor electrode material. *Key Eng. Mater. Trans. Tech. Publ.* **2017**, 756−763.
24. Wang, L.; Feng, X.; Ren, L.; Piao, Q.; Zhong, J.; Wang, Y.; Li, H.; Chen, Y.; Wang, B. Flexible solid-state supercapacitor based on a metal–organic framework interwoven by electrochemically-deposited PANI. *J. Am. Chem. Soc.* **2015**, *137*, 4920–4923.
25. Sundriyal, S.; Shrivastav, V.; Kaur, H.; Mishra, S.; Deep, A. High-performance symmetrical supercapacitor with a combination of a ZIF-67/rGO composite electrode and a redox additive electrolyte. *ACS Omega* **2018**, *3*, 17348−17358.
26. Yang, J.; Ma, Z.; Gao, W.; Wei, M. Layered structural Co-based MOF with conductive network frames as a new supercapacitor electrode. *Chem.: A Eur. J.* **2017**, *23*, 631-636.
27. Ran, F.; Xu, Pan, D.; Liu, Y.; Bai, Y.; Shao, L. Ultrathin 2D metal-organic framework nanosheets in situ interpenetrated by functional CNTs for hybrid energy storage device. *Nano-Micro Lett.* **2020**, *12*, 46.
